# Associations of sodium and potassium intake with chronic kidney disease in a prospective cohort study: findings from the Hispanic Community Health Study/Study of Latinos, 2008–2017

**DOI:** 10.1186/s12882-022-02754-2

**Published:** 2022-04-06

**Authors:** Samuel L. Swift, Yelena Drexler, Daniela Sotres-Alvarez, Leopoldo Raij, Maria M. Llabre, Neil Schneiderman, Linda Van Horn, James P. Lash, Yasmin Mossavar-Rahmani, Tali Elfassy

**Affiliations:** 1 Center for Healthcare Equity in Kidney Disease, University of New Mexico Health Science Center, Albuquerque, NM USA; 2 Katz Family Division of Nephrology and Hypertension, Department of Medicine, University of Miami Miller School of Medicine, Miami, USA; 3 Department of Biostatistics, University of North Carolina at Chapel Hill, Chapel Hill, NC USA; 4 Department of Psychology, University of Miami, Coral Gables, FL USA; 5 Department of Preventive Medicine, Feinberg School of Medicine, Northwestern University, Evanstown, IL USA; 6 Department of Medicine, University of Illinois College of Medicine, Chicago, IL USA; 7 Department of Epidemiology & Population Health, Albert Einstein College of Medicine, New York, NY USA

**Keywords:** Chronic kidney disease, Sodium, Potassium, Epidemiology, Cohort study

## Abstract

**Background:**

According to dietary recommendations, reduction of sodium intake has potential to reduce Chronic Kidney Disease (CKD) risk; however the role of dietary potassium and the sodium -to- potassium ratio in the development of CKD is unclear.

**Methods:**

We studied 9778 participants of the Hispanic Community Health Study/Study of Latinos (HCHS/SOL) from four US urban communities. Participants were aged 18–74 yrs., free from CKD at baseline in 2008–2011 and re-examined between 2014 and − 2017. Dietary intake of sodium, potassium and the ratio of dietary sodium -to- potassium were measured from two baseline 24-h dietary recalls. Incident CKD was defined as: 1) estimated glomerular filtration rate (eGFR) decline of 1 unit per year and eGFR < 60 ml/min/1.73m^2^ or 2) albumin to creatinine ratio ≥ 30 mg/g at the follow-up visit. We used multivariable survey weighted Poisson regression to estimate adjusted incident rates of incident CKD.

**Results:**

At baseline, mean age was 41 years. Average follow up time was 6.2 years. From fully adjusted Poisson regression analyses, self-reported sodium intake was not associated with incident CKD. However, for each 500 mg decrement in potassium intake, there was an 11% increase risk of incident CKD (IRR = 1.11, 95% CI = 1.00, 1.24). Additionally, every 1 M ratio increment of sodium -to -potassium ratio was associated with a 21% increased risk of incident CKD (IRR = 1.21, 95% CI = 1.02, 1.45), *p* < 0.05).

**Conclusions:**

We conclude that diets low in potassium and high in sodium are associated with increased risk of developing chronic kidney disease among healthy US Hispanic/Latino adults.

**Supplementary Information:**

The online version contains supplementary material available at 10.1186/s12882-022-02754-2.

## Introduction

Chronic kidney disease (CKD) is a major public health issue in the United States, with an estimated prevalence of over 10% [1] and growing [2]. Among US Hispanics/Latinos, the largest ethnic minority population, the prevalence of CKD is even higher at 13.7% [3]. The growing rates of CKD are concerning as CKD increases the risk of cardiovascular disease (CVD) and kidney failure [4], contributing greatly to health and other economic costs [5]. To help prevent CKD, dietary modification is an often recommended strategy [6]. Diets high in sodium are associated with hypertension and decreased kidney function [7–10]. Thus, sodium reduction is generally recommended as a lifestyle modification strategy for people diagnosed with CKD [11–14]. However, the benefits of a low sodium diet in those with kidney impairment remain somewhat controversial [15] and recommendations are not based on directly established relationships between high sodium and CKD [16].

To date, few studies have considered the role of dietary potassium in the relationship between sodium and kidney function [8, 17–21], and none, to our knowledge, in a diverse US Hispanic/Latino population. While there are many studies that have examined the relationship between dietary sodium and kidney function [8, 9, 17, 19, 22–27], most studies include only persons already diagnosed with CKD [9, 20, 21, 24–26], diabetes [28], or another chronic condition [17, 23–27]. Studies that have examined whether dietary sodium intake is associated with the development of CKD are sparse [18, 19, 22] and there is far less research [8, 17–21] that simultaneously examines the role of the sodium -to -potassium ratio with kidney function. Additionally, prior research shows that associations between dietary intakes of sodium and potassium and CKD may differ based on the presence of chronic conditions such as obesity, hypertension, or diabetes [8].

Despite having a high prevalence and incidence of CKD [3, 29], in addition to an unfavorable sodium and potassium profiles [30], the relationship between sodium and potassium with CKD has not previously been explored among diverse US Hispanics/Latinos [31]. The goal of this study is to address this gap by quantifying the association between sodium, potassium, and sodium -to- potassium ratio with incident CKD among Hispanics/Latinos living in the US.

## Methods

### Study population

The Hispanic Community Health Study/Study of Latinos (HCHS/SOL) is an ongoing population based prospective cohort study of 16,415 self-identified Hispanics/Latinos living in the United States. In brief, participants from diverse Hispanic/Latino heritage groups aged 18 to 74 years were recruited between 2008 and 2011, using a two-stage probability sampling design to oversample select Hispanic/Latino groups across study sites. These participants were recruited from areas in or in proximity to four urban field centers in Bronx, New York, Chicago, Illinois, Miami-Dade, Florida, and San Diego, California. Participants were re-examined at a second follow up examination that took place between years 2014 and 2017. During visits, participants underwent laboratory tests, physical examinations, and answered a series of survey questions in the language of preference. At each study site, informed consent was obtained from all participants. This study is in accordance with the principles of the Declaration of Helsinki. Protocols were approved by the Institutional Review Boards of all participating institutions. Further methodology of the study is described elsewhere [32]. The datasets generated and/or analyzed during the current study are not publicly available due to data security but are available from the corresponding author on reasonable request. These data are available upon request from the HCHS/SOL study via the study website at https://sites.cscc.unc.edu/hchs/StudyOverview.

### Measures

#### Sodium and potassium (exposures)

##### Self-reported intake

At baseline, bilingual registered dieticians collected two 24-h dietary recalls from participants [33]. The first recall was collected during the in-person clinic interview at baseline, and the second recall was collected via phone within the subsequent 3 months. A two-dimensional food model was shown to all participants to quantify portion sizes. The Nutrition Data System for Research (NDS-R) software developed by the University of Minnesota Nutrition Coordinating Center was used to collect dietary data using the multiple-pass method [34], to derive values for sodium (mg/day), and potassium (mg/day). The NDS-R software includes over 18,000 foods, 8000 brand-name products, including Hispanic foods. The two dietary recalls were then averaged, and 99% of the sample provided at least one dietary recall. Sodium -to- potassium ratio was calculated as the molar ratio of daily sodium (mmol) to daily potassium (mmol).

##### Calibrated intake

To account for measurement error associated with self-reporting usual sodium [35, 36] and to a lesser extent potassium intake [36], we derived secondary “calibrated intake” of sodium, potassium, and sodium -to- potassium-ratio [36]. Within 6 months to a year of participating in the HCHS/SOL, a sub-sample of 485 HCHS/SOL participants were recruited into the Study of Latinos Nutrition and Physical Activity Assessment Study (SOLNAS) which was designed to assess measurement error of energy, protein, sodium and potassium derived from dietary recall [37]. Described elsewhere [36], 24-h urine was collected to measure sodium and potassium biomarkers. Thus, SOLNAS participants had measures of sodium and potassium intake from 24-h dietary recalls (from the HCHS/SOL parent study) and from 24-h urinary recovery biomarkers (from the SOLNAS ancillary study). Using this subsample, calibration equations were developed by regressing log transformed urinary sodium biomarker on 24-h dietary recall derived sodium, adjusting for covariates [36]. We used the regression calibration equations to predict the nutrient intake for all HCHS/SOL participants. Because these are biomarker-calibrated values and not observed values, the SE of the diet-CKD model must be corrected to account for the extra uncertainty introduced by using calibration equations. To do so, we used the resampling-based multiple imputation to estimate the SE [38]. Specifically, we first generated 500 SOLNAS bootstrap samples of size *n* = 485 with replacement. Second, we fit the regression calibration equation on each bootstrap sample and estimated 500 sets of regression coefficients. Third, for each set of regression coefficients we obtained biomarker-calibrated sodium in the full HCHS/SOL sample. Fourth, we fit the model of interest for each of the 500 biomarker-calibrated nutrients. Lastly, we obtained the SE of the regression coefficients by accounting for the within bootstrap sample variability and the between bootstrap samples variability using Rubin’s rule. This process was repeated for potassium, and sodium -to- potassium-ratio.

#### Chronic kidney disease (outcome)

CKD was defined based on measures of estimated glomerular filtration rate (eGFR) and albumin to creatinine ratio (ACR). At both HCHS/SOL Visits 1 & 2, (V1 & V2) participants had fasting blood and urine samples collected. Serum cystatin C was measured from blood using the turbidimetric method [39] on a Roche Modular P Chemistry Analyzer (Gentian AS, Moss, Norway). Creatinine was also measured from serum and urine using the creatinase enzymatic method (Roche Diagnostics, Indianapolis, IN), on a Roche Modular P Chemistry Analyzer (Gentian AS, Moss, Norway). Using the CKD Epi cystatin C equation [40], eGFR (ml/min/1.73m^2^) was calculated for each participant from serum cystatin c, serum creatinine, sex, age, and race [40]. Albumin was measured from urine using the immunoturbidimetric method on the ProSpec nephelometric analyzer (Dade Behring GMBH, Marburg, Germany). For each participant, ACR (mg/g) was defined as the ratio of urinary albumin (mg/dL) to urinary creatinine (g/dL). Prevalent CKD was defined as either eGFR < 60 ml/min/1.73m^2^ or ACR ≥ 30 mg/g [29, 41]. As previously described in this cohort [29], incident CKD was then defined as meeting at least one of two conditions at the second examination: 1) eGFR < 60 ml/min/1.73m^2^ and eGFR decline ≥1 ml/min per year and/*or* 2) ACR ≥ 30 mg/g. Participants with prevalent CKD at the baseline examination were excluded from the analysis.

#### Other measures

Participants were asked to state their country of birth and select a category that best described their Hispanic/Latino heritage group including, “Central American,” “Cuban,” “Dominican,” “Mexican,” “Puerto-Rican,” “South American,” descent or “more than one group,” or “other”. Age, sex, years of education completed, health insurance status, years in the US, smoking status (never, current, or former smokers), alcohol use (number of drinks per week), and physical activity (METs/week) were all also obtained via questionnaire. Physical activity was assessed (METs/week) using a modified Global Physical Activity Questionnaire [42]. Body mass index (BMI, kg/m^2^) was assessed using measured height (m) and weight (kg). Systolic blood pressure measured at baseline was defined as the average of three repeat seated measurements obtained after a 5-min rest using the Omron HEM-907 blood pressure cuff [43]. Participants were asked to bring in all medications that they were currently taking at the baseline examination and these medications were scanned to define use of hypertensive medications, diuretics, and ACE-Inhibitors. Diabetes was defined as measured fasting plasma glucose ≥126 mg/dL, measured 2 h post-load glucose level ≥ 200 mg/dL, measured glycated hemoglobin (A1C) level ≥ 6.5%, or use of hypoglycemic agents at visit 1 (scanned medications) [44]. Finally, total cholesterol (mg/dL) was measured from fasting blood samples [45].

#### Analytical sample

From the 16,415 participants at baseline, 11,623 participants were present at V2. Given our interest in new onset or incident CKD, we excluded 1425 participants who had prevalent (*n* = 1331), or missing (*n* = 94) CKD information at V1 and also excluded 320 participants with missing information on CKD at V2. We further excluded 100 participants who did not have at least one dietary measure of sodium or potassium, resulting in a final analytical sample of 9778 participants.

### Statistical analysis

First, we provide descriptive statistics on demographic, socio-economic, behavioral, and clinical characteristics. Next, we estimated the age and sex-adjusted incidence rate of CKD according to unweighted tertiles of 24-h recall sodium, potassium, and sodium- to -potassium ratio. Using Poisson regression models to estimate incidence rate ratios, we assessed the associations between 500 mg increments of sodium, 500 mg decrements of potassium, and one unit increments of sodium -to -potassium ratio (mmol/mmol) with incident CKD. For each predictor of interest, we fit four sets of models. The first model was adjusted for demographic characteristics (age, sex, study site, and Hispanic/Latino heritage) along with follow-up time. In model two we additionally adjusted for socio-economic factors including: education, income, marital status, nativity/years in the US, and health insurance. In the third model we additionally adjusted for behavioral factors including: smoking, drinking, and self-reported physical activity. In model 4, we further adjusted for clinical factors including: BMI systolic blood pressure, hypertension medication, total cholesterol, and diabetes status. To ensure that the effects of each nutrient were independent of the other nutrient, in the models for sodium we adjusted for dietary potassium, the models for potassium were adjusted for dietary sodium. Next, to determine whether the associations between 24-h recall sodium, potassium, and sodium -to- potassium ratio were modified by either hypertension or diabetes, we included multiplicative interaction terms separately for all our final Poisson regression models for each predictor of interest. Significant interactions (*p* < 0.1 for interactions) resulted in stratified models [46].

Next, we repeated our main analyses using calibrated values of sodium, potassium, and sodium -to- potassium ratio. We also examined whether meeting the recommended dietary cutoffs for sodium (less than 2300 mg) [47] and potassium (greater than 4700 mg) [48] was associated with incident CKD. Finally, because there are medications that can interfere with urinary excretion of sodium and potassium [49, 50] (applicable to our calibrated measures only), for our calibrated measures, we conducted sensitivity analyses restricted to individuals 1) not taking diuretic medications and 2) not taking Angiotensin-converting enzyme (ACE). We also conducted two additional sensitivity analyses. To assess for the possibility of a nonlinear relationship between our exposures of interest and incident CKD, we repeated our fully adjusted models (model 4) using tertiles of each predictor (as we did for our unadjusted incidence rates). Finally, because we used the composite CKD outcome as defined above, we repeated our fully adjusted models (model 4) examining the relationship between each exposure of interest with incident decreased eGFR (< 60 ml/min/1.73 m2 with > 1 ml/min/1.73 m2 decline) and incident albuminuria (albumin to creatinine ratio ≥ 30 mg/g) as distinct outcomes.

All analyses were conducted using the R (version 4.0.2) or SAS (version 9.4) software. Analyses accounted for the complex survey design including clustering, stratification 2 sample weights which adjusted for the Visit 2 [32].

## Results

At baseline, among those free of CKD, the mean age was 41.1 years old and 51.5% were women (Table 1). Overall, 7.6% were of Central American, 20.0% of Cuban, 9.7% of Dominican, 38.0% of Mexican, 15.1% of Puerto Rican, and 5.0% of South American heritage. Most had an income less than $30,000 (61.5%), 31.0% has less than a high school education, 49.3% were married, 22.8% were born in the US, and 49.7% had health insurance. Similar proportions of participants can from the four study centers as follows: Bronx (27.7%), Chicago (16.1%), Miami (29.5%), and San Diego (26.6%). Of the health behaviors and clinical risk factors, 19.6% were smokers, and 68.2% met 2008 physical activity guidelines. On average, adults drank 5.3 drinks per week, had an average BMI of 29.4, and average systolic BP of 119.6 mmHg. About 10.7% of individuals were on hypertension medication. Mean total cholesterol was 194.7 mg/dl, and 12.0% had diabetes. Of our three exposures of interest, the mean intake per day was: 3203 mg for sodium. The mean intake per day of potassium was 2433 mg. Finally, we found that our sample had a mean a molar ratio of 2.4 for sodium -to- potassium ratio.

**Table 1:** Baseline (2008–2011) characteristics of participants free from chronic kidney disease in 2008–2011, Hispanic Community Health Study/Study of Latinos. (*n* = 9778)

	Unweighted N	% or mean	Standard error
**Socio-demographic Characteristics**			
Age, mean		41.1	0.3
Women	6163	51.5	0.7
Hispanic/Latino heritage			
Central American	1023	7.6	0.7
Cuban	1390	20.0	1.6
Dominican	854	9.7	0.7
Mexican	4098	38.0	1.6
Puerto Rican	1432	15.1	0.8
South American	704	5.0	0.8
Mixed/Other	268	4.5	0.4
Less than high school education	3561	31.0	0.8
Income < $30,000	6194	61.5	1.0
Married	5322	49.3	1.0
Nativity/years in the US			
Foreign born < 10 years in the US	2280	28.8	1.1
Foreign born, 10+ years in the US	5943	48.4	0.9
US born	1527	22.8	0.9
Health insured	4834	49.7	1.1
Study Site			
Bronx	2128	27.7	1.5
Chicago	2653	16.1	1.1
Miami	2428	29.5	2.1
San Diego	2569	26.6	1.7
**Health Behaviors**			
Current smoker	1734	19.6	0.8
Drinks per week, mean		5.3	0.2
Meets 2008 physical activity guidelines	6186	68.2	0.8
Sodium intake (recall) (mg/day), mean		3203.3	29.3
Potassium intake (recall) (mg/day), mean		2433.3	17.5
Sodium:Potassium (recall) (mmol/mmol), mean		2.4	0.0
**Clinical risk factors**			
BMI (kg/m^2^), mean		29.4	0.1
Systolic blood pressure (mmHg), mean		119.6	0.3
Hypertension medication	1473	10.7	0.4
ACE Inhibitors^a^	990	7.1	0.4
Diuretics	860	6.3	0.3
Total cholesterol (mg/dL), mean		194.7	0.6
Diabetes	1687	12.0	0.4

^a^ACE inhibitors referes to angiotensin-converting enzyme medications

**Fig. 1:**
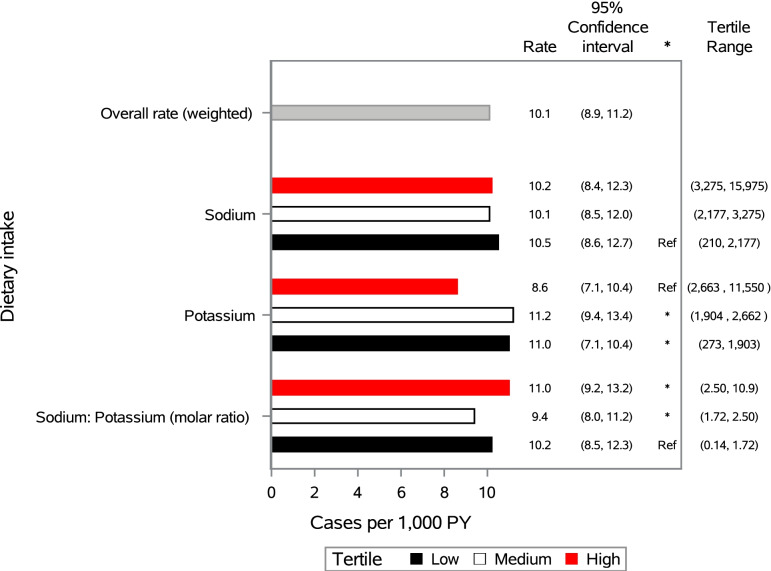
Age and sex adjusted incidence of chronic kidney disease^ǂ^ by tertile of sodium and potassium intake, The Hispanic Community Health Study/Study of Latinos, 2008–2017. ^ǂ^Incident chronic kidney disease is defined as eGFR < 60 ml/min/1.73 m2 and/or albumin to creatinine ratio ≥ 30 mg/g. PY refers to person years. *Rate significantly different than reference group *p* < 0.05

Over an average of 6.2 years of follow-up, the age and sex adjusted incidence of CKD was 10.1 per 1000 PYs (Fig. 1). The Incidence Rate (IR) did not differ by tertile of sodium intake. However, compared to the IR (IR = 8.6 per 1000 PY, 95% CI = 7.1, 10.4) in the highest tertile of potassium intake (Range = 2663 mg/day to 11,550 mg/day), those in the medium (Range = 1904 mg/day to 2662 mg/day), and low (273 mg/day to 1903 mg/day) tertile of potassium intake had a significantly higher IR (medium IR = 11.2 per 1000 PY, 95% CI = 9.4, 13.4; and low IR = 11.0 per 1000 PY, 95% CI = 7.1, 10.4). The rate (IR = 11.0 per 1000 PY, 95% CI = 9.2, 13.2) seen in the highest tertile of calibrated sodium -to- potassium ratio (range: 2.85 to 10.9), and the medium tertile (range = 1.72 to 2.50, IR = 9.4 per 1000 PY, 95% CI = 8.0, 11.2) were significantly higher compared to those in the lowest sodium -to- potassium ratio tertile (range = 0.14 to 1.72, IR = 10.2 per 1000 PY, 95% CI = 8.5, 12.3).

**Fig. 2:**
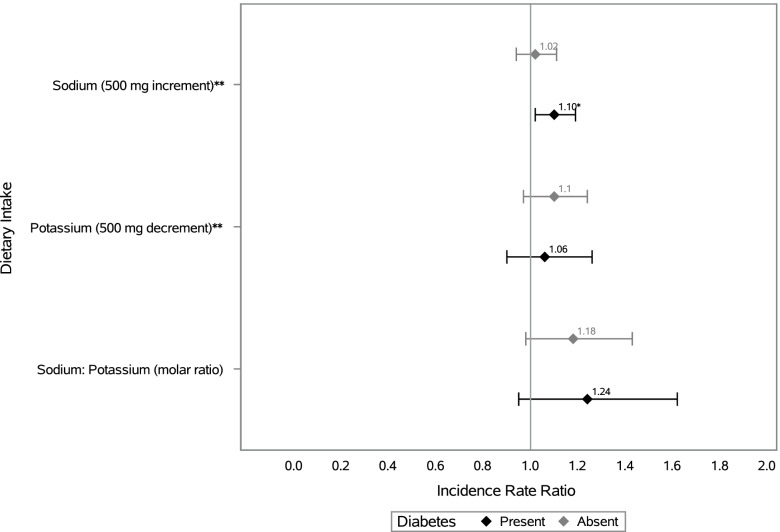
Incidence Density Ratios of chronic kidney disease^ǂ^ by sodium and potassium intake, stratified by diabetes status at baseline: The Hispanic Community Health Study/Study of Latinos, 2008–2017. ^ǂ^Incident chronic kidney disease is defined as eGFR < 60 ml/min/1.73 m2 and/or albumin to creatinine ratio ≥ 30 mg/g. *Incidence Densiity Ratio significant at *p* < 0.05. **P value for interaction < 0.10. ***All models are adjusted for: age, sex, time between visits, Hispanic/Latino heritage group, education, income, marital status, nativity/years in the US, health insurance, study site, smoking, drinking, physical activity, body mass index, systolic blood pressure, hypertension medication, and total cholesterol. The sodium model is additionally adjusted for potassium intake and the potassium model is additionally adjusted for sodium intake

In fully adjusted models each 500 mg increment of sodium failed to show a significant association with CKD (IRR = 1.05, 95% CI: 0.99, 1.12) (Table 2). However, from fully adjusted models, each 500 mg decrement of potassium was associated with an 11% increased risk of incident CKD (IRR = 1.11, 95% CI = 1.00, 1.24). Additionally, each molar increment in sodium -to- potassium ratio was associated with a 21% increased risk of CKD (IRR = 1.21, 95% CI = 1.04, 1.45). We tested whether the association between each 24-h recall exposure of interest and CKD differed based on: hypertension, diabetes, or obesity status and found statistically significant interactions (*p* < 0.10) for diabetes with sodium and potassium intake. Stratified results displayed in Fig. 2 showed that among those with diabetes, every 500 mg/day increment of sodium (from 24-h recall) was associated with a 9% increase in risk of CKD (IRR = 1.10, 95% CI = 1.02, 1.19), with no association detected among persons without diabetes. The interaction between diabetes and potassium intake was significant at *p* < 0.10, however the IRRs for the association between potassium intake and incident CKD did not achieve statistical significance at *p* < 0.05 when we stratified our models by diabetes status. In our analysis examining whether meeting recommended cutoffs for dietary intakes of these nutrients was associated with incident CKD, (Supplementary Table 1) neither the associations for sodium intake nor potassium intake were significant, although the association for potassium intake was similar in direction to those found in our main analysis. From our sensitivity analysis using tertiles of each nutrient, in fully adjusted models, compared with the lowest tertile of sodium -to- potassium ratio, tertile 2 was associated with a lower risk of CKD (IRR = 0.65, 95% CI = 0.43, 0.99), with no significant associations for tertile 3 compared with the lowest tertile (IRR = 1.21, 95% CI = 0.80, 1.84). In our sensitivity analysis examining the associations between each nutrient of interest with incident decreased eGFR and incident albuminuria (as distinct outcomes), we found that that greater sodium, lower potassium, and greater sodium to potassium ratio were associated with increased risk of albuminuria but not decreased eGFR.

**Table 2:** Associations between dietary sodium, potassium, and sodium to potassium ratio (2008-2011) with incident chronic kidney disease^a^ (2014–2017), Hispanic Community Health Study/Study of Latinos

	Model 1	Model 2	Model 3	Model 4
	Incidence Density Ratio (95% CI)	Incidence Density Ratio (95% CI)	Incidence Density Ratio (95% CI)	Incidence Density Ratio (95% CI)
Sodium (500 mg increment)	1.00 (0.97, 1.04)	1.01 (0.97, 1.05)	1.01 (0.96, 1.07)	1.05 (0.99, 1.12)
Potassium (500 mg decrement)	**1.05 (1.00, 1.10)**	1.04 (1.00, 1.09)	1.08 (0.99, 1.17)	**1.11 (1.00, 1.24)**
Sodium: Potassium (molar ratio)	**1.12 (1.00, 1.24)**	**1.11 (1.00, 1.22)**	**1.23 (1.05, 1.44)**	**1.21 (1.04, 1.42)**

^a^Incident chronic kidney disease is defined as eGFR < 60 ml/min/1.73 m2 with > 1 ml/min/1.73 m2 decline and/or albumin to creatinine ratio ≥ 30 mg/g. Model 1 is adjusted for: age, sex, time between visits, and Hispanic/Latino heritage group, with the potassium model adjusted for sodium intake and the sodium model is adjusted for potassium intake; Model 2 is additionally adjusted for: education, income, marital status, nativity/years in the US, study site, and health insurance; Model 3 is additionally adjusted for: smoking, drinking, and physical activity; Model 4 is additionally adjusted for: body mass index, systolic blood pressure, hypertension medication, total cholesterol, and diabetes

### Results using calibrated measures

Using biomarker calibrated nutrients, neither sodium, potassium, nor sodium -to- potassium ratio were statistically significantly associated with CKD (Table 3). However, the effect size for the sodium -to- potassium ratio was similar in direction and greater in magnitude to that using our 24-h recall measure of sodium -to- potassium ratio. In sensitivity analyses using our biomarker-calibrated nutrients restricted to individuals not on diuretics or not on ACEI (Supplementary Table 2), results were largely consistent in direction and magnitude compared with our final adjusted models, although not statistically significant.

**Table 3:** Associations between dietary sodium, potassium, and sodium (2008–2011) to potassium ratio with incident chronic kidney diseaseǂ (2014–2017), Hispanic Community Health Study/Study of Latinos, using nutrient calibration equations

	Incidence Density Ratio	95% CI
Sodium 500 mg^b^	1.02	(0.98, 1.06)
Potassium 500 mg decrement ^a^	1.05	(0.98, 1.11)
Sodium: Potassium (1 M ratio) ^b^	1.93	(0.61, 6.07)

^a^Incident chronic kidney disease is defined as eGFR < 60 ml/min/1.73 m2 with > 1 ml/min/1.73 m2 decline and/or albumin to creatinine ratio ≥ 30 mg/g. All models are adjusted for: age, sex, time between visits, Hispanic/Latino heritage group, education, income, marital status, nativity/years in the US, language preference, study site, health insurance, supplement use, smoking, drinking, physical activity, body mass index, systolic blood pressure, hypertension medication, total cholesterol, and diabetes. The potassium model is adjusted for sodium intake and the sodium model is adjusted for potassium intake. ^b^Models are additionally adjusted for overall resturant score and fast food resturant score

## Discussion

In a population-based cohort of diverse US Hispanics/Latinos followed for an average of 6 years, lower potassium and higher sodium -to- potassium ratio were associated with increased risk of CKD. Additionally, the association between sodium and CKD differed by diabetes status with higher sodium associated with greater risk of CKD among those with existing diabetes at baseline. Our results were particularly strong for sodium -to- potassium ratio which was associated with a 21% increased risk of CKD with every one-unit increment in the molar ratio. These results emphasize the importance of considering the ratio of sodium -to- potassium as a dietary factor in preventing the development of CKD among healthy adults.

Consistent with prior studies [18, 27], sodium alone was not a significant predictor of incident CKD in our main models. For example, among young participants of the Coronary Artery Disease In young Adults (CARDIA) study, mean 24-h sodium was not associated with the development of CKD over 20 years [17]. One potential explanation for these null results is the inability to accurately measure usual and habitual sodium intake—as diet varies over time [51]. Additionally, other research demonstrates that among persons with hypertension, a more complex nonlinear “U shaped” relationship between dietary sodium and CKD, in which both very high or very low sodium is associated with CKD [22]. For sodium and potassium, we did not find evidence of a nonlinear relationship with risk of CKD. However, for sodium -to- potassium ratio, we did find that risk of CKD was lowest in the middle tertile of sodium -to- potassium ratio suggesting the possibility of a “U shaped” association. Though we emphasize that these findings should be interpreted with caution and warrant further investigation. In our study, we found higher sodium to be associated with the development of CKD among individuals with pre-existing diabetes, but not persons without diabetes. This finding supports dietary recommendations urging individuals with diabetes to consider further sodium restriction to intake levels below the USDA recommendations [47]. In contrast, in another study conducted among individuals with diabetes and normal kidney function, lower potassium, but not higher sodium was associated with kidney and cardiovascular outcomes [28].

In our study, low potassium intake was associated with the development of CKD. This finding corroborates results from black and white young adult participants of the CARDIA study that showed that every 1 g increment of 24-h urinary potassium excretion was associated with a 29% reduction in risk of albuminuria in the subsequent 25 years [18]. However, in our calibrated models, we did not find an association between potassium and CKD. We propose that this may be explained by the variability added to the measured values through the calibration modeling process. Though our calibrated estimate for the association between potassium and CKD was stronger than our estimate using 24-h recall potassium (IDR = 1.20 vs IDR = 1.09), it did not achieve statistical significance due to the high variance (depicted with the wide confidence bands). Further examination of this important research question with more objective and repeated measurements may be required to fully elucidate the association between potassium and kidney function. For example, in research conducted in a population-based sample in Switzerland, high sodium and high sodium -to- potassium ratio but not low potassium was associated with greater decline in eGFR [19]. This is in contrast to our findings which did not show any independent associations between sodium, potassium, or the sodium -to- potassium ratio with decreased eGFR, though we caution against over-interpretation of these differences. We note that our study was conducted in a US Hispanic/Latino population with unique characteristics and with different measures of each nutrient of interest. Finally, we highlight that our general findings showing that higher sodium and higher sodium -to- potassium ratio, and lower potassium are associated with a worsening of kidney function are consistent with other studies conducted in population-based samples [18, 19]. Further, among individuals with CKD, there are a number of studies showing mixed findings with respect to the relationship between potassium intake and continued eGFR decline [20, 21]. For example, research from a clinic based retrospective cohort study of CKD patients in Japan showed that high potassium intake was associated with CKD progression [21]. However, research from the Chronic Renal Insufficiency Cohort (CRIC) study in the United States showed that among individuals with CKD, higher potassium intake was associated with slower eGFR decline [20]. We emphasize that our study was conducted in a population based sample free from CKD, and our findings are therefore most relevant for the prevention of CKD rather than the management of CKD.

To our knowledge, this is the first study to demonstrate associations between the ratio of dietary sodium -to- potassium intake and CKD in a population of Hispanics/Latinos who are relatively free from multiple comorbid conditions. Our research builds on previous evidence suggesting that the ratio of sodium -to- potassium intake may be an important predictor of CKD [8, 17, 18], eGFR decline [19] and other chronic diseases [52] in addition to dietary sodium [18, 19, 22] or potassium [17, 27] intake alone. Our results build on these findings by demonstrating this association with CKD among a population with most participants free of comorbid conditions. Our results are also consistent with a larger body of research demonstrating the importance of the sodium -to- potassium ratio with other chronic disease outcomes, such as hypertension [52, 53] and general CVD outcomes [54, 55]. Our results emphasize that diets such as the Dietary Approaches to Stop Hypertension Diet (DASH) [56], which have been demonstrated to reduce risk for CKD [57], continue to be important for CKD prevention because these diets simultaneously emphasize decreased sodium and increased potassium intake.

There are several mechanistic pathways by which low dietary potassium and high dietary sodium -to- potassium ratio may lead to decreased kidney function. Higher sodium intake is a risk factor for elevated blood pressure [58], and hypertension is in turn a risk factor for CKD [59]. Diets higher in potassium are thought to reduce vascular resistance [60, 61], lowering the risk for hypertension and also directly lowering the risk of kidney dysfunction by increasing eGFR [61, 62]. Diets high in potassium intake may also directly improve kidney function through increased kallikrein expression which has been demonstrated to reduce kidney injury in animal models [63].

This study is not without limitations. First, our primary dietary exposure measures were ascertained using two 24-h dietary recalls, which may be inaccurate [64]. However, we were able to collect two dietary recall surveys on 94% of participants, accounting for within person variability. Studies that have compared 24-h dietary recall to 24-h urine collection (the gold-standard assessment of sodium) [65] have found sodium to be underreported, particularly among those with higher BMI [64]. To address this limitation, we also included calibrated measures of sodium and potassium intake, which were designed to correct for this measurement error using data from 24 h urinary sodium and potassium excretion measurements taken from a subsample of HCHS/SOL participants [36]. In this analysis, we found an effect size similar in direction and stronger in magnitude to that of our primary measure for sodium -to- potassium ratio. However, these results were not significant, possibly due to the variability that calibration added to our models. Finally, our use of the composite CKD outcome makes it difficult to examine the relationships between each nutrient and incident decreased eGFR (< 60 ml/min/1.73 m2 with > 1 ml/min/1.73 m2 decline) or incident albuminuria (albumin to creatinine ratio ≥ 30 mg/g) independently. Our sensitivity analysis as seen in Supplementary Table 4 suggests that greater sodium, lower potassium, and greater sodium -to- potassium ratio are associated with albuminuria (albumin to creatinine ratio ≥ 30 mg/g) but not incident decreased eGFR (< 60 ml/min/1.73 m2 with > 1 ml/min/1.73 m2 decline). Though surprising, these results are consistent with other findings showing that sodium or potassium is associated with albuminuria but not decreased eGFR [18].

The relatively short six-year follow-up time in this cohort presents another limitation. CKD develops over decades throughout the life course and diet varies over time as well, due to changes in individual preferences in diet and secular changes in food availability [66]. We recommend that future studies examine this association in cohorts with repeated measures of dietary exposure and more follow-up time. We were unable to examine the associations between sodium and potassium with CKD according to clinically relevant cut points of sodium and potassium, making it harder to detect nonlinear relationships. Only 21.3 and 0.6% of HCHS/SOL participants met recommendations for sodium and potassium respectively [30]. Given this, sample sizes for individuals who met dietary recommendations were too small for meaningful comparisons. This limitation may be difficult to address in other US cohorts given that most of the US population fails to meet dietary guidelines for sodium or potassium [67]. Finally, there are some medications that can interfere with urinary excretion of sodium and potassium, thus contributing bias to our calibrated measures. To address these potential biases, we also conducted our sensitivity analyses among persons not taking diuretics or ACEI. In these analyses our results were consistent in magnitude and direction with our main analyses, however they were not statistically significant.

Our study also includes notable strengths. To our knowledge, associations between dietary intake of sodium, potassium, and CKD have not been demonstrated in a large sample of Hispanics/Latinos, so our findings have important implications for generalization to an understudied ethnic minority population. The large sample size of the HCHS/SOL study and the diversity of countries of origin strengthened our ability to detect associations that are both statistically significant and clinically meaningful. While previous research demonstrated the importance of the sodium -to- potassium-ratio for CKD among obese persons [8], and in a European population [19], our study builds on those findings by demonstrating this association among Hispanic persons living in the US with and without comorbid conditions.

In conclusion, our findings support dietary approaches to the prevention of CKD that focus simultaneously on limiting sodium and increasing potassium intake, rather than more simplistic approaches focused on individual nutrients. Potassium is a known marker of diets high in fruits and vegetables [68], so we believe our results further underscore the importance of diets high in fruits and vegetables in the prevention of chronic disease.

## Supplementary Information

**Additional file 1: Supplementary Table 1.** Associations between meeting recommended daily intake cutoff of dietary sodium, and potassium (2008–2011) with incident chronic kidney diseaseǂ (2014–2017), Hispanic Community Health Study/Study of Latinos, using nutrient calibration equations. **Supplementary Table 2.** Associations between dietary sodium, potassium, and sodium to potassium ratio (2008-2011) with incident chronic kidney disease^ǂ^ (2014–2017), Hispanic Community Health Study/Study of Latinos, among persons not on diuretics or ACE inhibitors. **Supplementary Table 3.** Associations between tertile of dietary sodium, potassium, and sodium to potassium ratio (2008-2011) with incident chronic kidney disease^ǂ^ (2014–2017), Hispanic Community Health Study/Study of Latinos. **Supplementary Table 4.** Associations between dietary sodium, potassium, and sodium to potassium ratio (2008-2011) with incident eGFR < 60 ml/min/1.73 m2 with > 1 ml/min/1.73 m2 decline or albumin to creatinine ratio ≥ 30 mg/g alone, (2014–2017), Hispanic Community Health Study/Study of Latinos.

## Data Availability

The data that support the findings of this study are available from Hispanic Community Health Study/ Study of Latinos but restrictions apply to the availability of these data, which were used under license for the current study, and so are not publicly available. Data are however available from the corresponding author Tali Elfassy upon reasonable request and with permission of Hispanic Community Health Study/ Study of Latinos. For more information please see: Study Overview | Hispanic Community Health Study / Study of Latinos (unc.edu).
