# Supplementary material for: Associations of sodium and potassium intake with chronic kidney disease in a prospective cohort study: findings from the Hispanic Community Health Study/Study of Latinos, 2008–2017
Source: BMC Nephrol. 2022 Apr 6;23:133. doi: 10.1186/s12882-022-02754-2 (PMC8988326; doi:10.1186/s12882-022-02754-2)
Supplement: Supplementary file 1 — Additional file 1: Supplementary Table 1. Associations between meeting recommended daily intake cutoff of dietary sodium, and potassium (2008–2011) with incident chronic kidney diseaseǂ (2014–2017), Hispanic Community Health Study/Study of Latinos, using nutrient calibration equations. Supplementary Table 2. Associations between dietary sodium, potassium, and sodium to potassium ratio (2008-2011) with incident chronic kidney diseaseǂ (2014–2017), Hispanic Community Health Study/Study of Latinos, among persons not on diuretics or ACE inhibitors. Supplementary Table 3. Associations between tertile of dietary sodium, potassium, and sodium to potassium ratio (2008-2011) with incident chronic kidney diseaseǂ (2014–2017), Hispanic Community Health Study/Study of Latinos. Supplementary Table 4. Associations between dietary sodium, potassium, and sodium to potassium ratio (2008-2011) with incident eGFR < 60 ml/min/1.73 m2 with > 1 ml/min/1.73 m2 decline or albumin to creatinine ratio ≥ 30 mg/g alone, (2014–2017), Hispanic Community Health Study/Study of Latinos. [file 12882_2022_2754_MOESM1_ESM.docx]

**Supplementary Table 1.** Associations between meeting recommended daily inake cutoff of dietary sodium, and potassium (2008-2011) with incident chronic kidney diseaseǂ (2014-2017), Hispanic Community Health Study/Study of Latinos, using nutrient calibration equations

|  | Incidence Density Ratio | 95% CI |  |
| --- | --- | --- | --- |
| Above daily sodium recommendations (>2300 mg) | 0.96 | (0.62, 1.48) |  |
| Below daily potassium recommendations (<4700 mg) | 1.09 | (0.40, 2.92) |  |

^ǂ^Incident chronic kidney disease is defined as eGFR < 60 ml/min/1.73m2 with >1ml/min/1.73m2 decline and/or albumin to creatinine ratio ≥ 30mg/g. All models are adjusted for: age, sex, time between visits, Hispanic/Latino heritage group, education, income, marital status, nativity/years in the US, language preference, study site, health insurance, supplement use, smoking, drinking, physical activity, body mass index, systolic blood pressure, hypertension medication, total cholesterol, and diabetes. The potassium model is adjusted for sodium intake and the sodium model is adjusted for potassium intake.

**Supplementary Table 2.** Associations between dietary sodium, potassium, and sodium (2008-2011) to potassium ratio with incident chronic kidney disease^ǂ^ (2014-2017), Hispanic Community Health Study/Study of Latinos, among persons not on diuretics or ACE inhibitors

|  | Not taking diuretics  (n=8,744) | | Not taking ACE* inhibitors  (n= 8,614) | |
| --- | --- | --- | --- | --- |
|  | Incidence Density Ratio | 95% CI | Incidence Density Ratio | 95% CI |
| Sodium 500 mg^ǂǂ^ | 1.01 | (0.97, 1.06) | 1.02 | (0.98, 1.07) |
| Potassium 500 mg decrement | 1.06 | (1.0, 1.26) | 1.05 | (0.98, 1.14) |
| Sodium: Potassium (1 molar ratio) ^ǂǂ^ | 2.12 | (0.63, 7.15) | 2.10 | (0.61, 7.19) |

^ǂ^Incident chronic kidney disease is defined as eGFR < 60 ml/min/1.73m2 with >1ml/min/1.73m2 decline and/or albumin to creatinine ratio ≥ 30mg/g. All models are adjusted for: age, sex, time between visits, Hispanic/Latino heritage group, education, income, marital status, nativity/years in the US, language preference, study site, health insurance, supplemeng use, smoking, drinking, physical activity, body mass index, systolic blood pressure, hypertension medication, total cholesterol, and diabetes. The potassium model is adjusted for sodium intake and the sodium model is adjusted for potassium intake. ^ǂǂ^Models are additionally adjusted for overall resturant score and fast food resturant score *ACE inhibitors referes to angiotensin-converting enzyme medications.

**Supplimentary table 3.** Associations between tertile of dietary sodium, potassium, and sodium (2008-2011) to potassium ratio with incident chronic kidney disease^ǂ^ (2014-2017), Hispanic Community Health Study/Study of Latinos.

|  | Incidence Density Ratio (95% CI) |
| --- | --- |
| Sodium first tertile | **ref** |
| Sodium second tertile | 1.01 (0.65, 1.58) |
| Sodium third tertile | 1.37 (0.76, 2.45) |
| Potassium first tertile | **1.72 (1.09, 2.72)** |
| Potassium second tertile | 1.63 (0.99, 2.67) |
| Potassium third tertile | **ref** |
| Sodium: Potassium (molar ratio) first tertile | **ref** |
| Sodium: Potassium (molar ratio) first tertile | **0.65 (0.43, 0.99)** |
| Sodium: Potassium (molar ratio) first tertile | 1.21 (0.80, 1.84) |

^ǂ^Incident chronic kidney disease is defined as eGFR < 60 ml/min/1.73m2 with >1ml/min/1.73m2 decline and/or albumin to creatinine ratio ≥ 30mg/g. All models are adjusted for: age, sex, time between visits, Hispanic/Latino heritage group, education, income, marital status, nativity/years in the US, language preference, study site, health insurance, supplement use, smoking, drinking, physical activity, body mass index, systolic blood pressure, hypertension medication, total cholesterol, and diabetes. The potassium model is adjusted for sodium intake and the sodium model is adjusted for potassium intake.

**Supplementary Table 4.** Associations between dietary sodium, potassium, and sodium (2008-2011) to potassium ratio with incident eGFR < 60 ml/min/1.73m2 with >1ml/min/1.73m2 decline or albumin to creatinine ratio ≥ 30mg/g alone. (2014-2017), Hispanic Community Health Study/Study of Latinos

|  | Incident eGFR ≤ 60 ml/min/1.73m2 with >1ml/min/1.73m2 decline | | Incident albumin to creatinine ratio ≥ 30mg/g | |
| --- | --- | --- | --- | --- |
|  | Incidence Density Ratio | 95% CI | Incidence Density Ratio | 95% CI |
| Sodium 500 mg | 0.89 | (0.77, 1.02) | **1.08** | **(1.00, 1.16)** |
| Potassium 500 mg decrement | 0.97 | (0.84, 1.13) | **1.14** | **(1.01, 1.27)** |
| Sodium: Potassium (1 molar ratio) | 0.88 | (0.61, 1.27) | **1.25** | **(1.06, 1.48)** |

All models are adjusted for: age, sex, time between visits, Hispanic/Latino heritage group, education, income, marital status, nativity/years in the US, study site, health insurance, smoking, drinking, physical activity, body mass index, systolic blood pressure, hypertension medication, total cholesterol, and diabetes. The potassium model is adjusted for sodium intake and the sodium model is adjusted for potassium intake.
